# A novel ferroptosis-related lncRNA signature for prognosis prediction in gastric cancer

**DOI:** 10.1186/s12885-021-08975-2

**Published:** 2021-11-13

**Authors:** Jianming Wei, Ye Zeng, Xibo Gao, Tong Liu

**Affiliations:** 1 Department of General Surgery, Tianjin Medical University General Hospital, Tianjin, China; 2 Department of Laboratory Medicine, Wuhan Children’s Hospital, Tongji Medical College, Huazhong University of Science and Technology, Wuhan, Hubei China; 3 Department of Dermatology, Tianjin Children’s Hospital, Tianjin, China

**Keywords:** Ferroptosis, Gastric cancer, Long non-coding RNA, Prognosis, Bioinformatics

## Abstract

**Background:**

Gastric cancer (GC) is a common malignant cancer with a poor prognosis. Ferroptosis has been shown to play crucial roles in GC development. Long non-coding RNAs (lncRNAs) is also associated with tumor progression in GC. This study aimed to screen the prognostic ferroptosis-related lncRNAs and to construct a prognostic risk model for GC.

**Methods:**

Ferroptosis-related lncRNAs from The Cancer Genome Atlas (TCGA) GC expression data was downloaded. First, single factor Cox proportional hazard regression analysis was used to select seven prognostic ferroptosis-related lncRNAs from TCGA database. And then, the selected lncRNAs were further included in the multivariate Cox proportional hazard regression analysis to establish the prognostic model. A nomogram was constructed to predict individual survival probability. Finally, we performed quantitative reverse transcription polymerase chain reaction (qRT-PCR) to verify the risk model.

**Results:**

We constructed a prognostic ferroptosis-related lncRNA signature in this study. Kaplan-Meier curve analysis revealed a significantly better prognosis for the low-risk group than for the high-risk group (*P* = 2.036e-05). Multivariate Cox proportional risk regression analysis demonstrated that risk score was an independent prognostic factor [hazard ratio (HR) = 1.798, 95% confidence interval (CI) =1.410–2.291, *P* < 0.001]. A nomogram, receiver operating characteristic curve, and principal component analysis were used to predict individual prognosis. Finally, the expression levels of AP003392.1, AC245041.2, AP001271.1, and BOLA3-AS1 in GC cell lines and normal cell lines were tested by qRT-PCR.

**Conclusions:**

This risk model was shown to be a novel method for predicting prognosis for GC patients.

**Supplementary Information:**

The online version contains supplementary material available at 10.1186/s12885-021-08975-2.

## Background

Gastric cancer (GC) is a common cancer. It was the third leading cause of cancer-related deaths in 2018 [1]. The incidence and mortality rates of GC have increased in China [2]. After surgery, multimodal therapies, including chemoradiation and chemotherapy, are utilized to prevent recurrence. Although such treatments have improved the survival rates of patients, patients with GC still have a survival rate of approximately 30% worldwide [3]. Therefore, novel biomarkers for GC need to be explored to predict the progression, prognosis, and response to treatment.

Ferroptosis is a non-apoptotic form of cell death [4]. Ferroptosis is associated with small molecules that target the antioxidant system or enzymes such as system xc − and glutathione peroxidase 4 (GPX4) [5]. Studies have shown that ferroptosis is associated with drug resistance [6, 7]. Zhang, H et al. found that ferroptosis promoted drug resistance in GC [8], while Shin, D. et al. indicated that Nrf2 inhibition reversed resistance to GPX4 inhibitor-induced ferroptosis in head and neck cancers [9]. These results revealed that ferroptosis played an important role in cancer development.

Long non-coding RNAs (lncRNAs) are a class of transcripts longer than 200 nucleotides [10]. Increasing studies have shown that lncRNAs play a crucial role in tumor progression [11, 12]. Recently, molecular risk signatures as prognostic predictors of cancer progression were investigated, especially lncRNA signatures [13, 14]. However, the prognostic value of ferroptosis-related lncRNAs signature in GC has not been systematically explored.

In this study, we constructed a molecular signature model comprising four prognostic ferroptosis-related lncRNAs and assessed their prognostic ability for GC. This study showed that the prognostic ferroptosis-related lncRNA signature was a reliable prognostic predictor for GC. Moreover, we explored the correlation between clinical factors and the four prognostic ferroptosis-related lncRNAs in this risk model. Finally, four lncRNAs (AP003392.1, AC245041.2, AP001271.1 and BOLA3-AS1) were validated in GC cell lines.

## Materials and methods

### Data acquisition

A search was performed for the word “ferroptosis” in MSigDB (http://www.gsea-msigdb.org/gsea/msigdb/index.jsp) to download ferroptosis-related genes. The original transcriptome sequencing dataset and GC-clinical characteristics were obtained from TCGA database (https://portal.gdc.cancer.gov/). The survival time of all patients was ≥30 days.

### Identification and correlation analysis

A total of 40 ferroptosis-related genes were downloaded from MSigDB v7.2. First, we selected all lncRNA expression data from TCGA database as shown in Table 1.

**Table 1:** Ferroptosis-related lncRNAs in TCGA database

LINC01235 AL357552.2 AC115618.2 AC026271.3 AC098869.2 DM1-AS AC116366.1 Z93930.2 AL353622.1 AC016735.1 OTUD6B-AS1 AL035071.1 AL117379.1 AC022211.2 AC104794.2 AC005899.6 LINC00106 AL139011.1 AC090114.2 AC009237.14 AC016737.1 AC005391.1 AC026368.1 AC024075.2 NUTM2A-AS1 ST20-AS1 AC020913.3 AC107027.3 MIR4435-2HG AL359715.3 AC022893.1 DNAJC9-AS1 RAET1E-AS1 PRR34-AS1 AC090192.2 AC104170.1 AL355802.2 OR2A1-AS1 FOXP4-AS1 AC127024.2 LINC01612 AC018653.3 AP001107.9 LINC01842 AL390198.1 AC016590.2 AC108062.1 AC015911.3 AL158166.2 TFAP2A-AS1 AC245100.7 AC008105.3 NR2F1-AS1 AC067852.2 AL078581.1 LINC02404 AC005520.2 EPB41L4A-DT AP003392.1 AP003352.1 AC107068.1 AC012181.2 AC100830.2 CCDC183-AS1 ZFAS1 AL117382.1 RHPN1-AS1 PTOV1-AS1 LINC00944 KMT2E-AS1 AL122035.1 AL391056.1 AC004009.1 AC119403.1 AC011247.2 AC245041.2 AL390066.1 AP000254.1 LINC02041 AC002558.3 LYRM4-AS1 AL365361.1 AL359182.1 AC012181.1 AL117382.2 AC008440.3 AC147067.2 AC018926.2 AC090425.1 AC022306.2 AP001271.1 BOLA3-AS1 LINC02449 AL031714.1 AC093752.3 GASAL1 SBF2-AS1 C10orf25 DLEU2 AC022509.3 DLG5-AS1 AC245128.3 AC009126.1 AC129510.1 AC139795.2 AC021218.1 AL139246.5 AL139120.1 AC087752.3 LINC02535 AC087741.1 AC090517.2 AC087588.2 AC132938.3 AC004241.3 AC015982.1 AC099518.2 ZBTB46-AS1 AC004540.2 AC073046.1 AC015922.2 AC009275.1 HIF1A-AS2 AC022706.1 ZBED5-AS1 AC091563.1 AL391422.4 AP006284.1 UBXN10-AS1 SND1-IT1 MIR600HG BBOX1-AS1 AC116407.2 AC006942.1 LINC01560 SNHG7 PIK3IP1-AS1 AL359962.1 AL391069.3 AC115102.1 LINC00513 AC097639.1 AC145207.5 CRNDE AL021707.6 C8orf49 MIATNB AL158166.1 AL392172.1 AC092720.1 GS1-124 K5.4 AL604028.1 AC010531.6 ZNF426-DT AF001548.2 TNFRSF10A-AS1 HOXA11-AS PART1	

Pearson correlation analysis was performed to determine the correlation between ferroptosis-related genes and all lncRNA expression data of the samples. Finally, ferroptosis-related lncRNAs were identified based on the Pearson correlation coefficient and *p* values (|Cor Pearson | > 0.4, *p* < 0.001). The correlation between ferroptosis-related lncRNAs and clinical characteristics was analyzed using the R package “ggpubr.”

### Prognostic risk signature construction

Single-variable Cox proportional risk regression analysis was performed to identify ferroptosis-related lncRNAs, which were significantly associated with OS in TCGA GC dataset. Multivariate Cox proportional risk regression analysis was then used to establish the prognostic model for GC. The results were plotted using a forest map with the R package “ggplot2.” The risk score for each patient was calculated using the risk formula: explncRNA1*coef lncRNA1 + explncRNA2*coef lncRNA2 + … + explncRNAi*coef lncRNAi [15]. Kaplan - Meier plotter was used to analyze the different OS times between the high-risk and low-risk groups using the R package “survival.”

### PCA

PCA is a widely used tool for dimensionality reduction and feature extraction in the computer vision field [16]. The R package “scatterplot3d” was used to assess potential differences between the high-risk and low-risk groups.

### Construction of nomogram

A nomogram was built according to all the independent prognostic factors using the R package “rms” (https://cran.rproject.org/web/packages/rms/index.html). A calibration plot curve analysis was performed to assess the consistency between the actual and predicted survival.

### Cell culture and qRT-PCR

Human gastric epithelial cell line (GES-1) and GC cell lines (MKN-45 and AGS) were obtained from Tianjin Createch Biotechnology Co. LTD (Tianjin, China). All cells were maintained in RPMI 1640 medium (Invitrogen, Carlsbad, CA, USA) supplemented with 1% penicillin-streptomycin (Invitrogen) and 10% fetal bovine serum (Invitrogen) at 37 °C and 5% ­ CO_2_. qRT-PCR was performed according to the method described earlier [17]. Total RNA was extracted using TRIzol reagent (Invitrogen) and synthesized into cDNA using M-MLV reverse transcriptase (TaKaRa Bio, Japan) following the manufacturer’s instructions. qRT-PCR was performed using SYBR Green assay (Roche, Switzerland). Glyceraldehyde 3-phosphate dehydrogenase (*GAPDH*) or U6 was utilized as an endogenous reference. The primers sequences are listed in Supplementary Table S1.

### Statistical analysis

All data were analyzed using the R software (R version: 3.6.1) and the following packages: “limma,” “survival,” “Pheatmap,” “ggpubr,” and “survivalROC.” The OS difference was determined using the Kaplan - Meier analysis method and log-rank test. Statistical significance was set at *p* < 0.05.

## Results

**Fig. 1:**
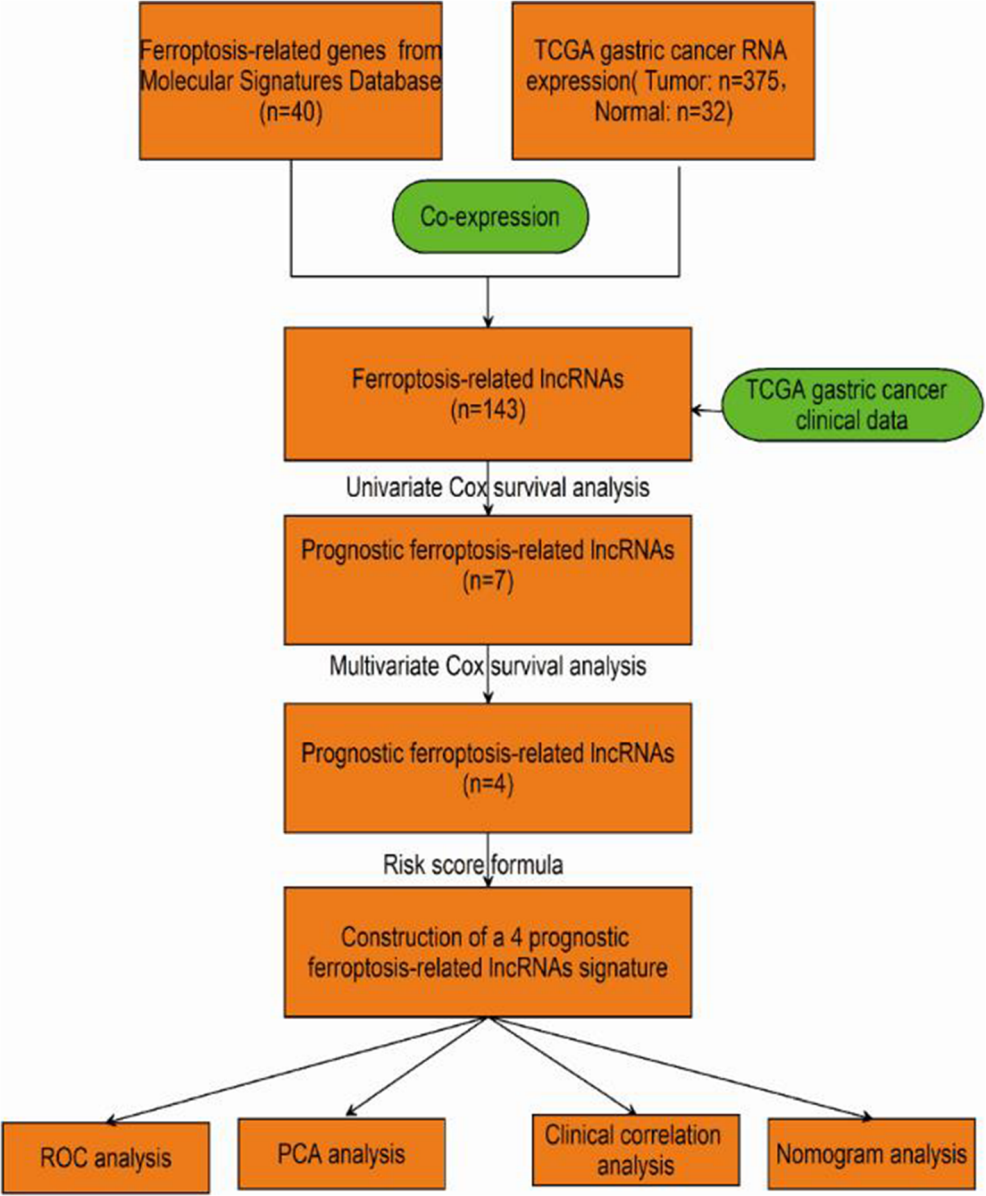
Workflow of the prognostic risk model analysis

The workflow of the prognostic model analysis is illustrated in Fig. 1. In this study, we used the data of 407 GC patients from The Cancer Genome Atlas (TCGA) cohort (T = 375, *N* = 32).

### Ferroptosis-related lncRNA identification in TCGA cohort

Forty ferroptosis-related mRNAs were selected based on one ferroptosis gene set (WP_FERROPTOSIS) from the Molecular Signatures Database (MSigDB). A total of 173 ferroptosis-related lncRNAs were screened out, with 148 ferroptosis-related lncRNAs positively correlated and 25 ferroptosis-related lncRNAs negatively correlated with GC according to the co-expression observed in TCGA cohort.

### Construction of prognostic ferroptosis -related lncRNA signature

**Fig. 2:**
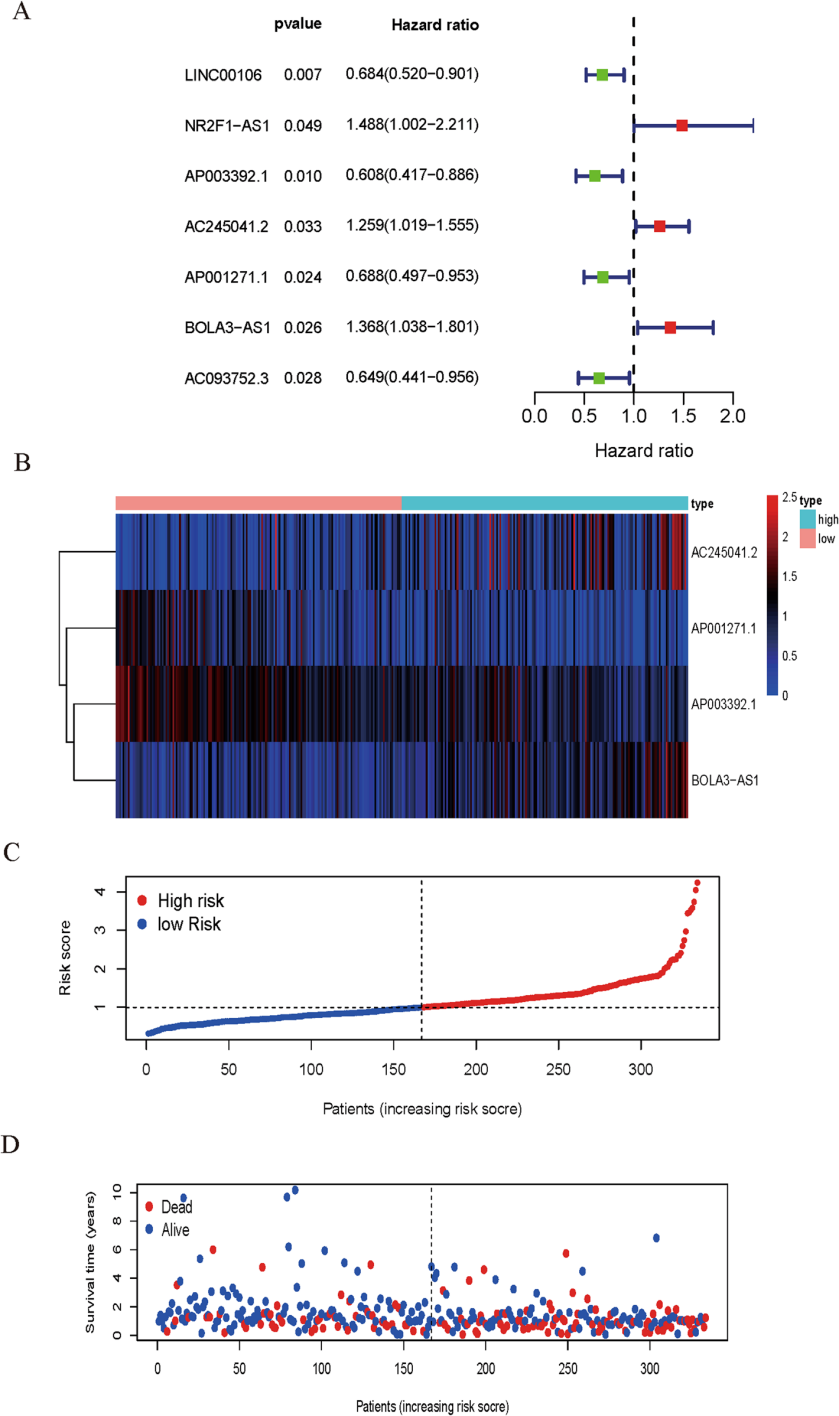
Outcomes of the four ferroptosis-related-lncRNA model in all the samples. (**A**) HR and 95% CI of the seven top lncRNAs using univariate Cox regression. (**B**) The distribution of the four ferroptosis-related lncRNA expression profiles of patients in all samples. (**C**) The distributions of the risk scores in all samples. (**D**) The distribution of the follow-up time in all samples

The clinical data of 334 GC patients were analyzed. Using univariate Cox regression analysis, seven prognostic ferroptosis-related lncRNAs were identified. The results were presented as a forest plot in Fig. 2A. Seven prognostic ferroptosis-related lncRNAs were further analyzed using multivariate Cox regression analysis. Finally, a 4-ferroptosis-related lncRNA signature model was established. The coefficients of each lncRNA were listed in Table 2.

**Table 2:** The coefficients (coef) of the prognostic four ferroptosis -related lncRNAs

id	coef	HR	HR.95 L	HR.95H	*p*value
AP003392.1	−0.50932	0.600904	0.40146	0.899432	0.013322
AC245041.2	0.235579	1.265641	1.02113	1.568701	0.031486
AP001271.1	−0.31336	0.73099	0.511205	1.045267	0.085919
BOLA3-AS1	0.525606	1.691484	1.267559	2.257188	0.000356

The risk score was calculated as follows: Risk score = (− 0.509319782 × expression value of AP003392.1) + (0.235578881 × expression value of AC245041.2) + (− 0.313355736 × expression value of AP001271.1) + (0.525606422 × expression value of BOLA3-AS1). Heatmap library was used to evaluate the values of the risk scores. Based on the median risk score, we divided the patients into high-risk and low-risk groups and assessed the score’s ability. The patients were ranked from low to high according to the risk score. We have shown the population follow-up time and gene heat-map by ranking as well (Fig. 2B **-** D).

### Correlation between the four lncRNAs and clinical features

**Fig. 3:**
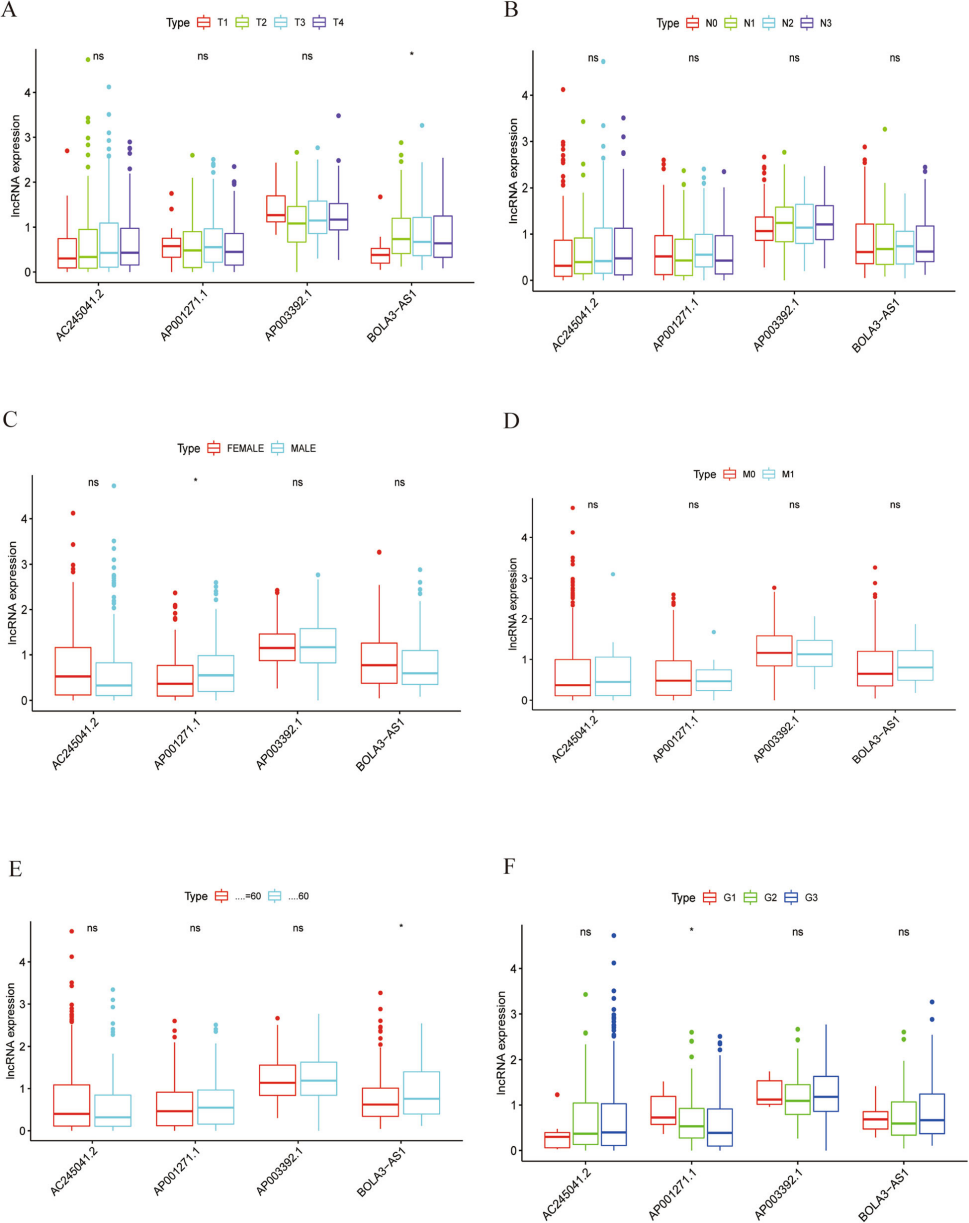
Correlation between the four ferroptosis-related lncRNAs and clinical features. The relationship between the expression of the four ferroptosis-related lncRNAs and (**A**) T, (**B**) N, (**C**) gender, (**D**) M, (**E**) age, and (**F**) grade. NS: Not Significant, *: *P* < 0.05. Note: T: Tumor, classified into T1, T2, T3, T4; N: Node, classified into N1, N2, N3; M: metastasis, classified into M0, M1

The correlation between the four prognostic ferroptosis-related lncRNA signatures and clinicopathological characteristics was assessed. The expression of BOLA3-AS1 was significantly associated with T and age (Fig. 3A, E). Moreover, AP001271.1 expression was significantly associated with gender and grade (Fig. 3C, F). However, the expression of none of the four lncRNAs was significantly associated with N and M (Fig. 3B, D).

### Univariate and multivariate cox regression analyses of the ferroptosis-related lncRNA signature for GC

**Fig. 4:**
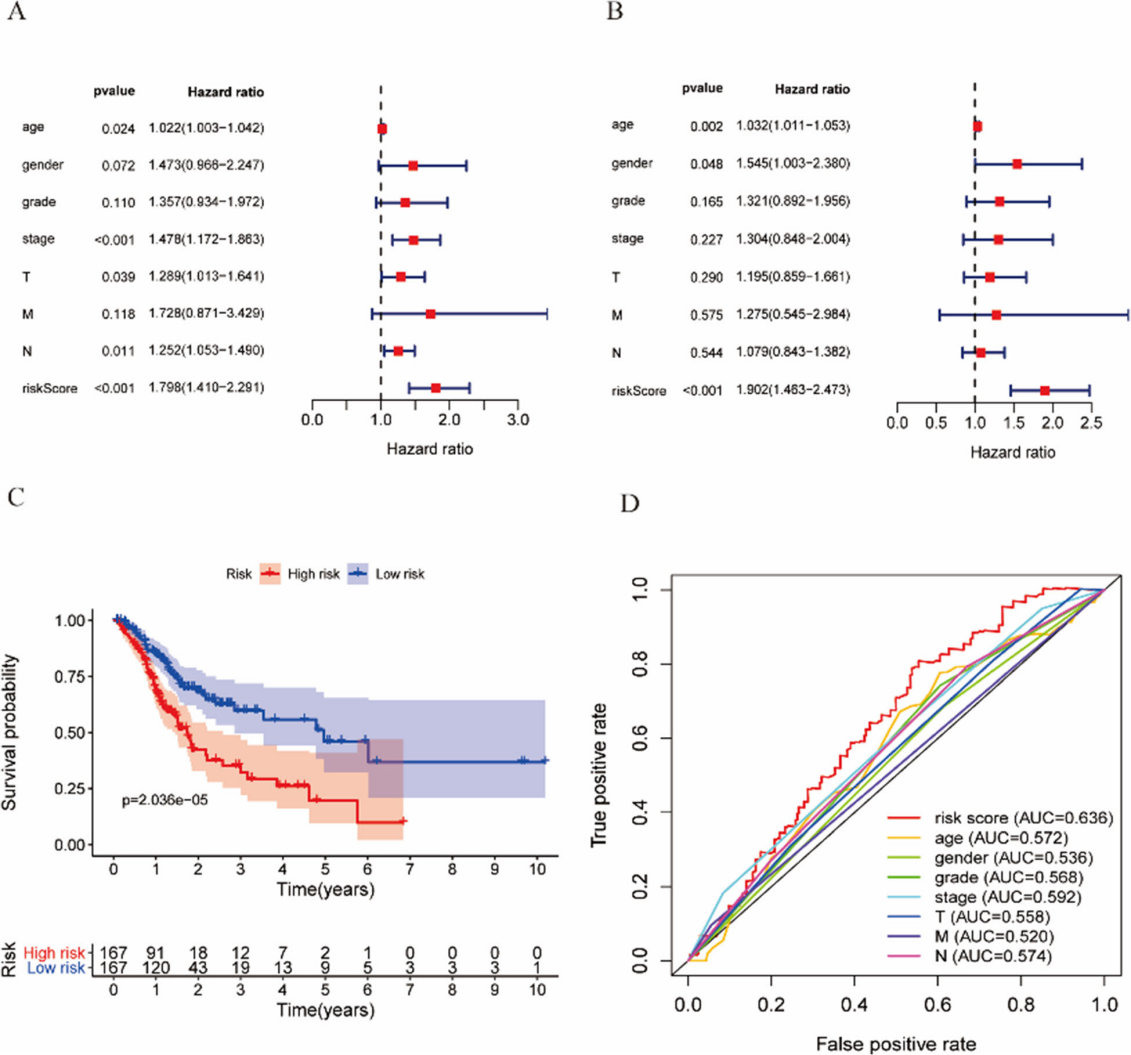
Univariate and multiple regression analysis of the ferroptosis-related lncRNA signature for GC. Results of the (**A**) univariate and (**B**) Multivariate Cox regression analysis show the effects of clinical factors and risk score in all samples. Results of the (**C**) survival analysis show the prognosis of high-risk and low-risk patients. (**D**) The ROC for risk-score, age, grade, stage, T, N, and M with OS for GC cohorts

In the univariate Cox regression analysis, age, stage, T, N, and risk score were significantly associated with overall survival (OS; HR = 1.022, 95% CI = 1.003–1.042, *P* = 0.024; HR = 1.478, 95% CI = 1.172–1.863, *P* < 0.001; HR = 1.289, 95% CI = 1.013–1.641, *P* = 0.039; HR = 1.252, 95% CI = 1.053–1.490, *P* = 0.011; 0.001; HR = 1.798, 95% CI =1.410–2.291, *P* < 0.001; respectively, Fig. 4A). In the multivariate Cox regression analysis, risk score was an independent predictor of OS (HR =1.902, 95% CI = 1.463–2.473, *P* < 0.001; Fig. 4B). The survival curve shows that the low-risk group has a better survival period than that of the high-risk group (Fig. 4C). In this study, the AUC of the ROC curve was calculated for risk score (AUC = 0.636), age (AUC = 0.572), gender (AUC = 0.536), grade (AUC = 0.568), stage (AUC = 0.592), T (AUC = 0.558), M (AUC = 0.520), and N (AUC = 0.574). The AUC value of the risk score was higher than that of other clinical parameters, revealing the good predictive power of the Cox regression model for predicting the prognosis of GC patients (Fig. 4D).

### Differential analysis between high-risk and low-risk groups

**Fig. 5:**
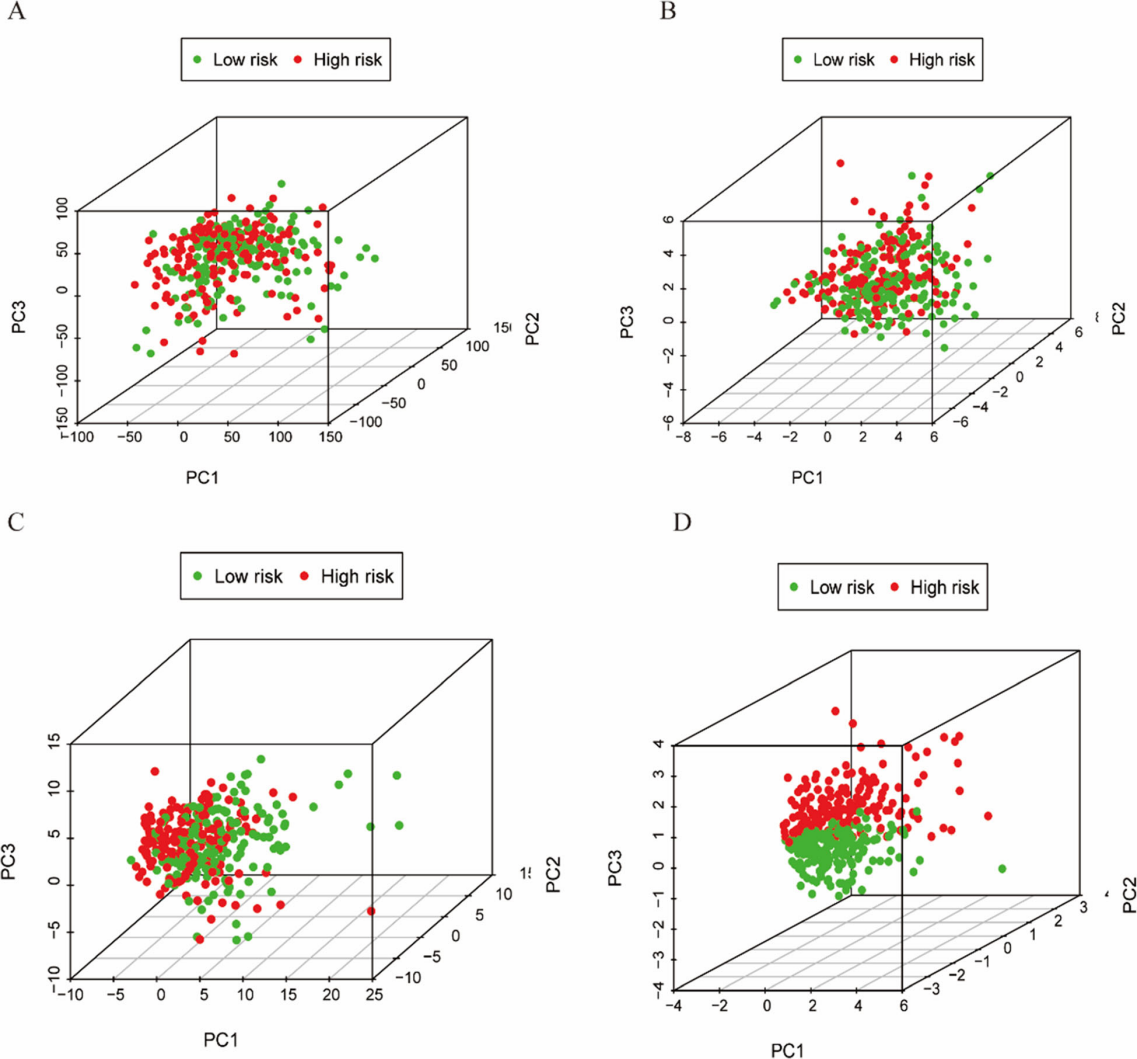
Principal component analysis. Results of the principal component analysis between low-risk and high-risk groups based on the expression of all genes (**A**), ferroptosis-related genes (**B**), and lncRNAs (**C**), and the four lncRNAs of the prognostic model (**D**)

Results of principal component analysis (PCA) are shown in Fig. 5. There were no significant differences between the high-risk and low-risk groups in the expression of all examined genes (Fig. 5A), and ferroptosis-related genes (Fig. 5B), and lncRNAs (Fig. 5C); however, but there was a significant difference between the high-risk and low-risk groups in the expression of for the four lncRNAs (Fig. 5D) used in the prognostic model. This study revealed that the high-risk and low-risk groups were significantly different in terms of the ferroptosis signature.

### Construction of nomogram

**Fig. 6:**
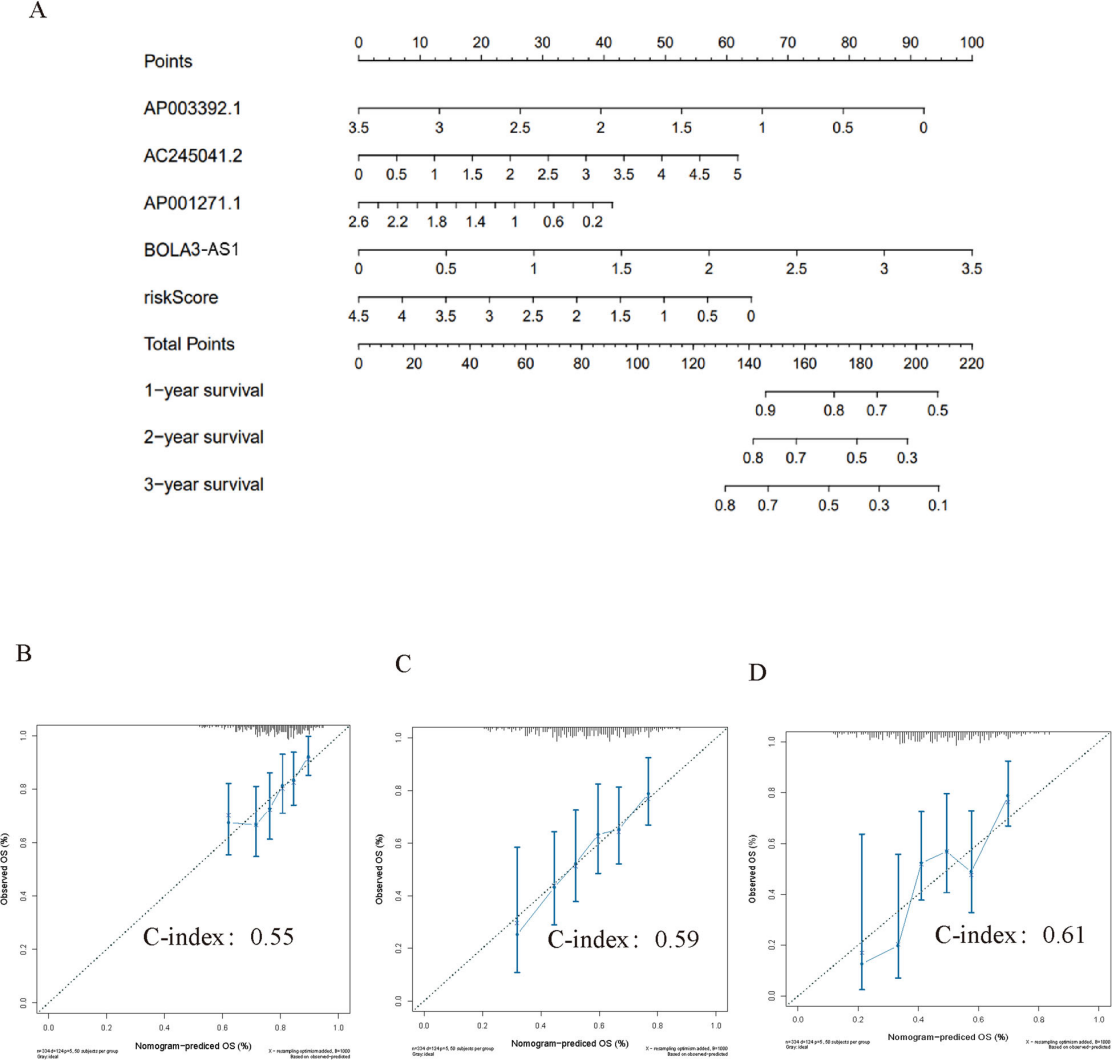
An individualized prediction model for determining the overall survival (OS) of GC patients. (**A**) Nomogram construction for predicting the 1-, 2- and 3- year OS of GC patients. (**B** - **D**) Calibration curve analysis

A nomogram was constructed to quantify the risk of each patient [18]. The nomogram was then used to predict the 1-year-, 2-year-, and 3-year OS by weighing AP003392.1, AC245041.2, AP001271.1, BOLA3-AS1, and the risk scores. The score assigned to each factor was proportional to its risk contribution to survival (Fig. 6A). The indications on the calibration curve matched well (Fig. 6B **-** D). The C-index of 1-year-, 2-year-, and 3-year OS was 0.55, 0.59, 0.61.

### The expression of AP003392.1, AC245041.2, AP001271.1, and BOLA3-AS1 in GC

**Fig. 7:**
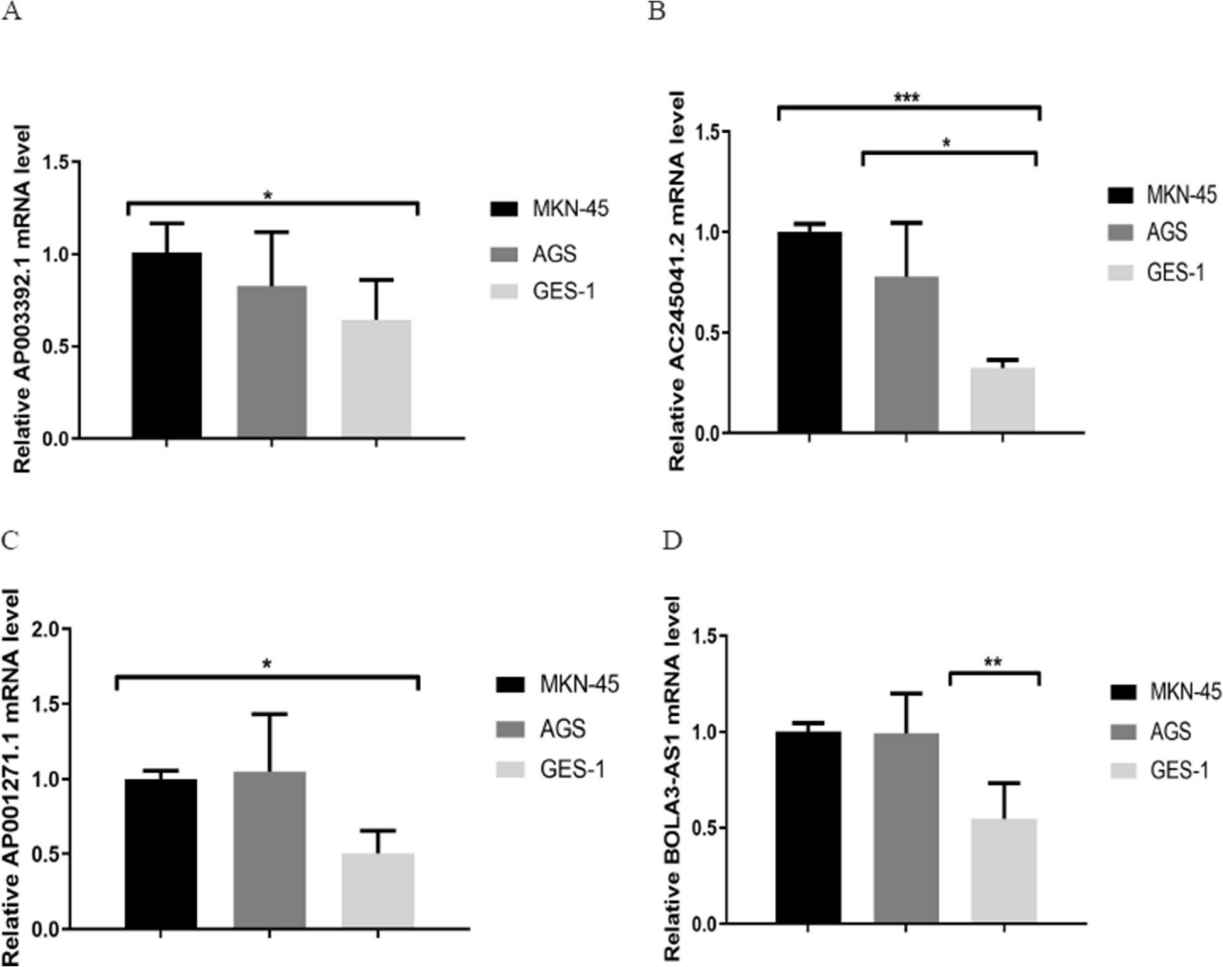
The expression of AP003392.1, AC245041.2, AP001271.1, and BOLA3-AS1 in GC cell lines

To further explore the expression of AP003392.1, AC245041.2, AP001271.1 and BOLA3-AS1, GC cell lines (MKN-45 and AGS) and human gastric epithelial cell lines (GES-1) were used to validate the expression levels of the four lncRNAs. Quantitative real-time PCR (qRT-PCR) analysis results showed that AP003392.1, AC245041.2, AP001271.1, and BOLA3-AS1 were differentially expressed in GC cell lines compared to that in gastric normal cell lines (Fig. 7). Moreover, these results showed that AP003392.1, AC245041.2, AP001271.1, and BOLA3-AS1 may play an important role in GC.

## Discussion

The term ferroptosis was coined in 2012 to describe iron-dependent cell death [19]. Ferroptosis is increasingly recognized to be associated with the prognosis of patients with GC and other cancers [20–25]. Several studies have focused on the effect of ferroptosis on tumor development and treatment. Carbone, M. et al. found that regulating ferroptosis could be a new therapeutic approach in ovarian cancer [26], revealing the potential value of ferroptosis in guiding clinical decisions. Zhang et al. showed that ferroptosis was influenced by the differential regulation of transcription in liver cancer [27].

Recently, prognostic signatures, comprising mRNAs [28, 29], mRNA expression-based stemness index (mRNAsi) [30, 31], and microRNAs [32, 33] have been used to infer the prognosis of cancers. LncRNA signatures have been constructed to predict OS in many cancers [34–37]. Li, J. et al. identified a five-lncRNA signature that predicted the risk of tumor recurrence in breast cancer patients [38]. Zhu, X. et al. showed that long non-coding RNA signature could improve prognosis prediction in GC [39].

Previous studies have demonstrated that lncRNAs are closely related to ferroptosis [40–42]. Yang, Y et al. revealed that lncRNA ZFAS1 promoted ferroptosis via the miR-150-5p/SLC38A1 axis [43]. Wang et al. showed that nuclear lncRNA LINC00618 accelerates ferroptosis in a manner dependent on apoptosis [44]. However, the prognostic signature of ferroptosis-related lncRNAs in GC has rarely been explored.

In this study, we first identified 173 ferroptosis-related lncRNAs, and then, 7 prognostic ferroptosis-related lncRNAs were further analyzed using multivariate Cox regression analysis. Finally, a four -ferroptosis-related lncRNA signature model was established from TCGA datasets. We also demonstrated that four ferroptosis-related lncRNA signature was an independent risk factor for GC. This result indicated that the prognostic signature of the four ferroptosis-related lncRNAs could accurately predict the prognosis of GC patients.

Nomograms are widely used in prognostic prediction in oncology and medicine [45]. In the current study, a nomogram was constructed using AP003392.1, AC245041.2, AP001271.1, BOLA3-AS1, and the risk score. The nomogram showed a good performance in predicting 1-year, 2-year, and 3-year survival OS of GC patients, which may contribute to the promotion of individualized treatment of GC patients. To investigate the relationship between the four ferroptosis-related lncRNAs and clinical factors, we further analyzed the relationship between clinical features and the four lncRNAs. We found that the four prognostic ferroptosis-related lncRNAs were associated with T-staging, sex, age, and grade.

Interestingly, Wang, S et al. built a novel prognostic nomogram based on five lnc-RNAs in clear cell renal cell carcinoma, comprising AC026992.2, AC245041.2, LINC00524, LINC01956, and LINC02080 [46]. Previous work on lncRNA profiling also revealed that BOLA3-AS1 was associated with higher-risk myelodysplastic syndrome, which played an important role in the development of blood lineages such as platelets, erythrocytes, and myeloid cells [47]. However, the roles of AP003392.1 and AP001271.1 are yet unknown. To explore the expression levels of AP003392.1, AC245041.2, AP001271.1, and BOLA3-AS1, qRT-PCR analysis was applied. We found that the expression of AP003392.1, AC245041.2, AP001271.1, and BOLA3-AS1 was upregulated in GC cells lines. Thus, despite the important prognostic value of ferroptosis-related lncRNA signature identified in this study, future experiments on lncRNAs components are required to elucidate their roles in GC.

In conclusion, we demonstrated the prognostic value of a ferroptosis-related lncRNA signature, comprising AP003392.1, AC245041.2, AP001271.1, and BOLA3-AS1, thus providing a theoretical basis for ferroptosis-related targeted therapies.

Our study has some limitations. First, this was a retrospective study, and therefore it lacked novel clinical samples and data. Second, the mechanism and interrelationship among ferroptosis-related lncRNAs comprising this signature require further study. In conclusion, this study constructed a prognostic risk model consisting of four ferroptosis-related lncRNAs by analyzing the RNA-sequencing-based gene expression profiles of GC from TCGA database. This risk model has been established be independently associated with OS and facilitated the prediction of GC prognosis. However, the mechanisms underlying the interplay between ferroptosis and lncRNAs in GC require further investigation.

## Conclusions

This risk model was shown to be a novel method for predicting prognosis for GC patients. Thus, the mechanisms underlying the interplay between ferroptosis and lncRNAs in GC require further investigation.

## Supplementary Information

**Additional file 1.** The primers sequences of four ferroptosis-related lncRNAs.

## Data Availability

The data supporting the findings of this study are available in the supplementary material of this article.
