# Supplementary material for: A novel ferroptosis-related lncRNA signature for prognosis prediction in gastric cancer
Source: BMC Cancer. 2021 Nov 13;21:1221. doi: 10.1186/s12885-021-08975-2 (PMC8590758; doi:10.1186/s12885-021-08975-2)
Supplement: Supplementary file 1 — Additional file 1. The primers sequences of four ferroptosis-related lncRNAs. [file 12885_2021_8975_MOESM1_ESM.docx]

**The primers sequences:**

**AC245041.2**

| **Forward primer** | CCCAGGGTAGTTTTGCCTGT |  |  |  |  |  |  |  |  |
| --- | --- | --- | --- | --- | --- | --- | --- | --- | --- |
| **Reverse primer** | GTCCTGGCTGTGAGTGCTAA |  |  |  |  |  |  |  |  |

**AP001271.1**

| **Forward primer** | GGGCAAATGCAGGAAGCAAA |  |  |  |  |  |  |  |  |
| --- | --- | --- | --- | --- | --- | --- | --- | --- | --- |
| **Reverse primer** | TGGTTTCCCCAAAAGCACCT |  |  |  |  |  |  |  |  |

**AP003392.1**

| **Forward primer** | GAATTCACCCACCTCAGCC |  |  |  |  |  |  |  |  |
| --- | --- | --- | --- | --- | --- | --- | --- | --- | --- |
| **Reverse primer** | GTGTGCGTTTTCCCACTGTC |  |  |  |  |  |  |  |  |

**BOLA3-AS1**

| **Forward primer** | ACCAGTGTGCTACAGACTTC | 2.00 | |  |  |  |  |  |  |  |
| --- | --- | --- | --- | --- | --- | --- | --- | --- | --- | --- |
| **Reverse primer** | AGCCCCTAAGGATTTTAAAACACAA |  |  | |  |  |  |  |  |  |
|  |  |  |  | |  |  |  |  |  |  |
